# A Phase II study of trabectedin single agent in patients with recurrent ovarian cancer previously treated with platinum-based regimens

**DOI:** 10.1038/sj.bjc.6604088

**Published:** 2007-11-13

**Authors:** C N Krasner, D S McMeekin, S Chan, P S Braly, F G Renshaw, S Kaye, D M Provencher, S Campos, M E Gore

**Affiliations:** 1 Department of Medical Oncology, Massachusetts General Hospital, Boston, MA, USA; 2 Department of Gynecologic Oncology, University of Oklahoma Health Science Center, Oklahoma City, OK, USA; 3 Department of Clinical Oncology, Nottingham City Hospital, Nottingham, UK; 4 Department of Gynecologic Oncology, Hematology and Oncology Specialists, New Orleans, LA, USA; 5 Oncology Therapeutics, Johnson & Johnson Pharmaceutical Research & Development, L.L.C., Raritan, NJ, USA; 6 Department of Medicine, Royal Marsden Hospital, London, UK; 7 Department of Gynecologic Oncology, CHUM, Hôpital Notre-Dame, Montreal, Canada; 8 Department of Medical Oncology, Dana Farber Cancer Institute, Boston, MA, USA

**Keywords:** trabectedin, ovarian cancer, overall response rate, platinum-sensitive, platinum-resistant, progression-free survival

## Abstract

The objective of this study was to determine the objective response rate in patients with platinum-sensitive and platinum-resistant recurrent ovarian cancer to treatment with trabectedin (Yondelis®) administered as a 3-h infusion weekly for 3 weeks of a 4-week cycle. We carried out a multicentre Phase II trial of trabectedin in patients with advanced recurrent ovarian cancer. Trabectedin (0.58 mg m^−2^) was administered via a central line, after premedication with dexamethasone, to 147 patients as a 3-h infusion weekly for 3 weeks followed by 1-week rest. Major eligibility criteria included measurable relapsed advanced ovarian cancer and not more than two prior platinum-containing regimens. Patients were stratified according to the treatment-free interval (TFI) between having either platinum-sensitive (⩾6 months TFI) or platinum-resistant disease (<6 months TFI)/platinum-refractory disease (progression during first line therapy). In the platinum-sensitive cohort, 62 evaluable patients with measurable disease had an overall response rate (ORR) of 29.0% (95% CI: 18.2–41.9%) and median progression-free survival (PFS) was 5.1 months (95% CI: 2.8–6.2). Four patients with measurable disease per Response Evaluation Criteria in Solid Tumours (RECIST) criteria had no follow-up scans at the end of treatment. In the platinum-resistant/refractory cohort, 79 patients were evaluable with an ORR of 6.3% (95% CI: 2.1–14.2%). Median PFS was 2.0 months (95% CI: 1.7–3.5 months). Two patients with measurable disease per RECIST criteria had no follow-up scans at the end of treatment. The most frequent (⩾2% of patients) drug-related treatment-emergent grade 3/4 adverse events were reversible liver alanine transferase elevation (10%), neutropaenia (8%), nausea, vomiting, and fatigue (5% each). Trabectedin is an active treatment, with documented responses in patients with platinum sensitive advanced relapsed ovarian cancer, and has a manageable toxicity profile.

Ovarian cancer is often diagnosed in advanced stages due to the absence of overt signs or symptoms in earlier-stage disease, with poor long-term prognosis and 5-year survival rates <30% (American Cancer Society, 2005). Standard therapy is cytoreductive surgery followed by platinum-based chemotherapy. Despite high initial response rates, relapse and chemo-resistance eventually occur in the majority of patients with advanced disease (Markman and Bookman, 2000; Harper, 2002).

The National Institute of Health and Clinical Excellence (NICE) of United Kingdom guidelines (Technology Appraisal Guidance, 2005) recommend that patients with recurrent ovarian cancer should be classified by their duration of the response to initial platinum therapy: those with disease recurrence ⩾12 months after initial therapy are regarded as platinum-sensitive, whereas those with recurrence 6 to >12 months after initial therapy are regarded as partial platinum-sensitive (Gordon *et al*, 2001). Women who recur ⩽6 months after initial therapy are considered platinum-resistant and are less likely to receive further benefit from platinum. Women with disease progression during initial platinum therapy are regarded as platinum-refractory and require no further platinum therapy.

Few effective single agents are available for second-line and subsequent therapy following progression on platinum and taxanes (Markman and Bookman, 2000; Gordon *et al*, 2001). Current single agent chemotherapy options for patients include: re-treatment with a platinum-based regimen or single agent pegylated liposomal doxorubicin, topotecan, or gemcitabine, which have shown response rates in the range of 16–40% (Gordon *et al*, 2001; Markman *et al*, 2001; Garcia *et al*, 2004; Papadimitriou *et al*, 2004; National Comprehensive Cancer Network, 2005; Rose, 2005).

Trabectedin (ET-743, Yondelis®, PharmaMar, SA, Madrid, Spain and Johnson & Johnson Pharmaceutical Research & Development, LLC, Raritan, NJ, USA) is a tris, tetrahydroisoquinoline alkaloid isolated originally from the marine ascidian *Ecteinascidia turbinate* and currently produced synthetically. It binds to the minor groove of DNA at the N2 position of guanine, inducing a bend towards the major groove (Pommier *et al*, 1996; Zewail-Foote and Hurley, 1999; van Kesteren *et al*, 2003). Trabectedin inhibits transcription of heat shock-inducible genes (Minuzzo *et al*, 2000) and interacts with the transcription-coupled nucleotide excision repair (TC-NER) system, resulting in formation of lethal DNA strands, cell cycle arrest, and apoptosis by a process that is p53 independent (Erba *et al*, 2001; Takebayashi *et al*, 2001). *In vitro*, low concentrations (2–80 nM) of trabectedin disrupt progression of human cancer cells through S phase, resulting in accumulation in late S and G_2_/M (Martinez *et al*, 2001; Mandola *et al*, 2005). Recent data also suggest that the enzyme poly ADP-ribose polymerase (PARP) may contribute to the cellular sensitivity of trabectedin (Mandola *et al*, 2005).

Trabectedin has shown *in vitro* cytotoxicity against melanoma, ovarian, colorectal, breast, brain, and lung cancer cell lines (Jimeno *et al*, 1996). Soft-tissue sarcoma cell lines have proven to be highly sensitive to growth inhibition in picomolar concentrations (Li *et al*, 2001). Similarly, growth in human solid tumour colony-forming assays was inhibited (Izbicka *et al*, 1998). Trabectedin also inhibits the development of human tumour xenografts in mice, including melanoma, ovarian cancer, non-small cell lung cancer, breast, and renal cell carcinomas, with cures in some models (Jimeno *et al*, 1996; Valoti *et al*, 1998; Hendriks *et al*, 1999). Valoti *et al*, (1998) found trabectedin active against xenografts, inducing long-lasting tumour regressions both in early and established tumours. Of note, activity was observed in ovarian xenograft tumours that are sensitive to Cisplatin as well as those which are resistant to cisplatin.

In Phase I studies, trabectedin showed activity in patients with ovarian cancer (Salazar *et al*, 2006), mesothelioma (Ryan *et al*, 2001), leiomyosarcoma (Villalona-Calero *et al*, 2002), melanoma (Ryan *et al*, 2001), breast cancer (Taamma *et al*, 2001), endometrial carcinoma (McMeekin *et al*, 2004), liposarcoma (Taamma *et al*, 2001), osteosarcoma (Taamma *et al*, 2001), and soft-tissue sarcomas (Yovine *et al*, 2004). Doses evaluated included 1- to 72-h infusions administered every 3 weeks, 1-h infusion daily for 5 days every 3 weeks, and a 3-h infusion once weekly for 3 out of 4 weeks (Ryan *et al*, 2001; Villalona-Calero *et al*, 2002; Twelves *et al*, 2003). The once weekly regimen for 3 out of 4 weeks appeared to provide antitumour activity with the benefit of improved patient tolerability and convenience of administration. Therefore, the current protocol was initiated at the time of the onset of initial Phase II studies.

A Phase II study conducted by the Southern Europe New Drugs Organization Foundation (SENDO) evaluated the use of trabectedin in 59 patients with advanced disease including refractory or relapsing ovarian cancer following a platinum-taxane regimen (Sessa *et al*, 2005). An initial dose of 1.65 mg m^−2^ (reduced to 1.3 mg m^−2^) was administered as a 3-h infusion every 3 weeks. An overall response rate (ORR) of 43.5%, one complete response (CR) and nine partial responses (PR), was reported in 23 evaluable platinum-sensitive patients. The duration of the patient who achieved a CR was 8.7 months. The patients who achieved PR had a median time to progression (TTP) of 7.9 months, and an additional nine patients exhibited stable disease (SD). The 28 patients with evaluable platinum resistance yielded an ORR of 7.1%, 2 patients with PR each with a TTP of 4 and 4.6 months. Stable disease was reported in eight (28.6%) additional patients. The 1.3 mg m^−2^ dose was well tolerated with transient increase in aminotransferases and non-fatal (G3) neutropaenia.

The present Phase II trial evaluated trabectedin as a single agent administered weekly for 3 of 4 weeks in patients with advanced relapsed ovarian cancer previously treated with a platinum-based regimen. This study was initiated when the appropriate schedule and dosing of trabectedin had not been determined. The primary objective was to determine the ORR in patients with platinum-sensitive and -resistant disease. Secondary objectives included duration of response, TTP, progression-free survival (PFS), overall survival (OS), and safety.

## MATERIALS AND METHODS

### Patient population

Eligibility criteria included histologically proven epithelial ovarian carcinoma, fallopian tube carcinoma, or primary peritoneal carcinoma (excluding peritoneal mesothelioma) treated with one to two prior platinum-containing regimens (if the second was started following a progression-free interval of ⩾6 months after the first regimen); age ⩾18 years; Eastern Cooperative Oncology Group (ECOG) performance status of 0 or 1; at least 1 measurable lesion according to Response Evaluation Criteria in Solid Tumours (RECIST) (Therasse *et al*, 2000) guidelines; adequate haematologic (haemoglobin >9.0 g per 100 ml, absolute neutrophil count (ANC) >1500 per μl, platelets ⩾100 000 per μl), renal (serum creatinine⩽ULN), and hepatic function (total bilirubin⩽ULN, total alkaline phosphatase⩽ULN (or if>ULN, alkaline phosphatase liver fraction or 5′-nucleotidase⩽ULN), AST and ALT⩽2.5 × ULN, and albumin⩾2.5 g l^−1^). The patient population in this study included platinum-sensitive, -resistant, and -refractory patients receiving second or third line therapy.

Exclusion criteria included prior trabectedin exposure; one prior non-platinum-containing regimen in platinum-sensitive patients (more specific exclusion added after a protocol amendment); more than two prior chemotherapy regimens; <4 weeks from radiation therapy hormonal therapy, biological therapy, any investigational agent; peripheral neuropathy⩾grade 2; history of other neoplastic disease (unless remission for >5 years); known CNS metastasis or other serious illness; and pregnant or lactating women or those of childbearing potential not employing adequate contraception.

The Independent Ethics Committee/Independent Review Board of each participating site approved the study. All patients gave written informed consent.

### Study design and evaluations

This multicentre, open-label, single-arm Phase II trial enrolled patients in two prospectively defined cohorts: a platinum-sensitive cohort, defined as relapse after a disease-free interval ⩾6 months from the end of the last platinum-based chemotherapy; or a platinum-resistant cohort, defined as disease progression <6 months from the end of the last platinum-based treatment. A 2-stage design was used to minimise exposure to dose and schedule of trabectedin until tolerability and efficacy were established.

Vital signs, haematologic parameters, blood chemistry, liver panels, and physical examinations were performed at baseline (within 14 days before administration of first drug dose) and throughout the study including treatment termination. Disease evaluations were made per RECIST guidelines (Therasse *et al*, 2000) utilising either CT or MRI. CA 125 levels were measured at screening, after every two cycles, and at study termination. Patients without measurable disease, clinical progression, or study discontinuations without radiology documentation at the end of the study were considered non-evaluable for response.

### Dosage and administration

Trabectedin was diluted in 500 ml of saline and administered via a central venous catheter. This resulted in two patients with tissue extravasations due to catheter-related malfunctions. A dose of 0.58 mg m^−2^ was administered once weekly as a 3-h infusion for 3 weeks, on a 28-day cycle. All patients were premedicated with 10 mg dexamethasone IV 30 min before trabectedin infusion, since dexamethasone pretreatment has been shown in animal and clinical studies to ameliorate the hepatotoxic effects of trabectedin by diminishing hepatocellular exposure to the drug (Puchalski *et al*, 2002; Sarah *et al*, 2003). Treatment was continued as long as clinical benefit was derived or until disease progression. Trabectedin was discontinued after patients received at least two cycles beyond a confirmed CR (McMeekin *et al*, 2005).

### Dose reductions and discontinuations

A maximum of two dose reductions (from 0.58. to 0.49 then to 0.40 mg m^−2^) were permitted based on toxicity. Prophylactic use of haematopoietic colony-stimulating factors was not permitted in the first cycle. In subsequent cycles, they could be used for secondary prophylaxis according to institutional and American Society of Clinical Oncology guidelines (American Society of Clinical Oncology). Treatment delays up to 3 weeks were permitted for persistent toxicity; beyond this, therapy was discontinued. Patients were withdrawn because of serious adverse events, disease progression, or consent withdrawal.

### Response and toxicity assessment

Tumour assessment was performed at baseline, every other treatment cycle until disease progression, and at the end of treatment. After completion of the treatment phase, patients were followed for survival every 3 months until death or a predetermined cutoff date approximately 2 months from last patient treatment termination. Adverse events were assessed at each treatment visit, treatment termination, and during follow-up. Toxicities were graded according to the National Cancer Institute Common Toxicity Criteria V2.0 (National Cancer Institute).

### Statistical methods

Sample size for each group was determined using a Simon 2-stage design. For the platinum-sensitive group, 23 patients were enrolled in the first stage. If at least four responders were observed, approximately 25 additional patients were enrolled until 48 platinum-sensitive patients were evaluable for response per RECIST guidelines. This design had 80% power to reject a response rate of 15% at a 5% significance level when the true response rate was 30%. In the platinum-resistant group, 30 patients were enrolled in the first stage. If at least two responders were observed, approximately 22 additional patients were enrolled until 52 platinum-resistant patients were evaluable for response per RECIST guidelines. This design had 80% power to reject a response rate of 5% at a 5% significance level when the true response rate was 15%.

For efficacy results, patients were further classified into subgroups based on TTP from initial platinum therapy: platinum-sensitive recurrence ⩾12 months after initial platinum-based therapy; partial-platinum-sensitive recurrence 6 to <12 months after initial therapy; platinum-resistant recurrence ⩽6 months after initial therapy; platinum-refractory, progression during initial platinum therapy. For final intent-to-treat efficacy analysis, response rate, TTP, PFS, OS, the 28 patients whose platinum sensitivity status on enrolment was unknown on initial review, but later resolved, were appropriately added back to either the platinum-sensitive or -resistant/refractory patient populations.

Prognostic factors including baseline ECOG performance status scores; number of platinum-based lines of therapy before enrolment; TTP from the start of last dose of chemotherapy, histology; histology grade; or CA125 levels at baseline were analysed for correlation with ORR.

Summaries of safety were based on laboratory data and treatment-emergent adverse events (TEAE), defined as any adverse event occurring on or after the first dose of trabectedin and within 30 days after the last dose.

## RESULTS

### Patient characteristics

Between October 2002 and September 2004, a total of 170 patients were screened for enrolment. Baseline characteristics for treated patients were similar for the platinum-sensitive and -resistant cohorts, with the exception of ECOG performance status (Table 1). Of the screened patients, 147 were part of the intent-to-treat population (23 patients were screen failures) and 141 were evaluable for efficacy per RECIST guidelines at the end of study treatment (Table 2). Nearly, all patients received prior cytoreductive surgery (98%), and 5% had previous radiotherapy. All patients had received prior platinum-containing chemotherapy. In the platinum-sensitive patient cohort, 74% had 1 prior line and 26% had 2 prior lines. Median time from the last chemotherapy regimen to the first dose was 11 months. In the platinum-resistant/refractory patient cohort, 65% had 1 prior line and 35% had 2 prior lines, median time from the last chemotherapy regimen to the first dose was 4.1 months.

### Treatment

The 66 platinum-sensitive patients received 304 cycles with a median of four cycles per patient (range, 1–11); median treatment duration was 18.6. weeks (range, 4.0–77); median cumulative dose was 6.5 mg m^−2^ (range, 1–15); and median dose intensity was 0.353 mg m^−2^ per week (range, 0.17–0.44) with a relative dose intensity of 81.1% (range, 38–102%). For the 81 platinum-resistant/refractory patients, 284 total cycles were administered with a median of two cycles (range, 1.0–22.0); treatment duration was 8.9 weeks (range, 4.0–98.0), median cumulative dose was 3.5 mg m^−2^ (range, 1.0–37.0), median dose intensity was 0.391 mg m^−2^ per week (range, 0.14–0.46), and the relative dose intensity was 90.0% (range, 32–105%).

For the platinum-sensitive patients, 42% had at least one cycle delay, 45% had at least one dose withheld, and 42% had at least one dose reduction. In the platinum-resistant/refractory patient cohort, 28% had at least one cycle delay, 20% had at least one dose withheld, and 23% had at least one dose reduction. Reasons for dose delays and reductions in both cohorts were divided equally between liver transaminase elevations and myelosuppression. Twenty-one of 66 (31.8%) patients in the platinum-sensitive cohort and 8 of 79 (10.1%) in the platinum-resistant cohort remained on therapy >6 months.

### Efficacy

Efficacy and survival data are shown in Table 3. Both statistical response targets (for platinum-sensitive and -resistant patients) were reached in our study. The 62 evaluable patients in the platinum-sensitive cohort had an ORR of 29.0% (27.3% of the total platinum-sensitive population) (4CR, 14PR) (95% CI: 18.2–41.9%). Partial platinum-sensitive patients (*n*=39) yielded an ORR of 25.6% (1CR, 9PR); SD in 13 (33.3%); disease control (CR+PR+SD) in 23 (60.0%); and disease progression in 16 (40%) patients. The platinum-sensitive subgroup (*n*=23) had an ORR of 34.8% (3CR, 5PR); SD in 9 (39.1%).

The 79 evaluable patients in the platinum-resistant/refractory patient cohort had an ORR of 6.3% (6.2% of the total platinum-resistant/refractory population) (5PR) (95% CI: 2.1–14.2%). The platinum-resistant subgroup (*n*=42) had an ORR of 4.8% (2PR), SD in 21 (50.0%), and disease progression in 19 (45.2%) patients. The platinum-refractory subgroup (*n*=37), ORR was 8.1% (3PR), SD in 15 (40.5%), and disease progression in 19 (51.3%) patients. Six patients of the total patient population were not evaluable because of early death, clinical progression, or early discontinuation unrelated to toxicity.

### Secondary efficacy end points

Secondary efficacy end points included time to response, duration of response, PFS, and OS. In the platinum-sensitive cohort, the partial platinum-sensitive subgroup had a median time to response of 1.8 months (range, 2–5); median duration of response, 5.2 months (95% CI: 3.7–5.8); and a median PFS of 4.0 months (95% CI: 1.7–6.1). In the platinum-sensitive subgroup (>12 month treatment-free interval), the median time to response was 1.9 months (range, 2–5); median duration of response was not estimable (NE) because very few patients had disease progression; and median PFS was 5.1 months (95% CI: 3.1–6.5) (Table 3).

The platinum-resistant subgroup (including platinum-resistant/refractory patient cohort) had a median time to response of 2.5 months (range, 2–3); median duration of response, 4.2 months; and a median PFS of 2.3 months (95% CI: 1.7–4.1). The platinum-refractory subgroup had median time to response of 5.6 months (range, 2–12); median duration of response, 11.0 months; and a median PFS of 1.9 months (95% CI: 1.6–2.6) (Table 3).

In the overall platinum-sensitive cohort, TTP was 5.2 months (95% CI: 3.1–6.5 months) and PFS was 5.1 months (95% CI: 2.8–6.2 months). Overall survival was NE because very few patients have died, with a 1-year survival rate of 55.9%. The overall platinum-resistant cohort had a TTP of 2.0 months (95% CI: 1.77–3.5 months), PFS of 2.0 months (95% CI: 1.7–3.5 months), and OS of 10.7 months with a 1-year survival rate of 24.5% (Table 3).

No statistically significant correlation was noted between the prognostic factors analysed and ORR, PFS, and OS, which may be explained, in part, by the small sample size.

The concurrence rate between CA125 response (defined as either a decrease of at least 50% in value or normalisation, confirmed after at least 28 days) and objective response was 80% in platinum-sensitive patients and 90% in platinum-resistant patients. The concurrence rate between CA125 response and objective response, including SD (>4 month) patients, was decreased to 77% in platinum-sensitive patients and 69% in platinum-resistant patients.

### Toxicity

Of the 147 patients treated, 146 (99%) reported at least one laboratory abnormality (worst CTC grade) while on treatment. A drug-related grade 3/4 TEAE was reported for 39% of the treated subjects, and in 14% of cycles of therapy. The most common worst grade 3/4 laboratory abnormalities reported were elevated ALT (12%/0%), hyponatraemia (8%/1%), neutropaenia (7%/1%), and hypoalbuminaemia (7%/0%) (Table 4).

The most common TEAEs causing dose modifications were elevated ALT (20%), granulocytopaenia (18%), increased alkaline phosphatase (16%), and infusion site complications (7%). Drug-related TEAEs that led to discontinuation of treatment were reported for 11 (7%) patients.

All 147 treated patients were included for safety analysis. Most commonly reported drug-related grade 3/4 TEAEs occurring in ⩾2% of evaluable patients were elevated ALT (11%), granulocytopaenia (6%), and nausea, vomiting, and fatigue (5% each). Incidence of alopecia was 9% with no drug-related grade 3/4 events. The most frequently drug-related TEAEs (grades 1–4) were nausea (67%), vomiting (56%), fatigue (60%), constipation (33%), abdominal pain (11%), and anorexia (28% each) (Table 5).

Five subjects died during study treatment or within 30 days of the last dose of the study drug. Disease progression was reported as the cause of death for two subjects, and death was attributed to drug-related TEAEs for one subject due to drug-related grade 3 dyspnoea, pulmonary oedema, central chest pain, heart murmur, bilateral pleural effusion, pulmonary hypertension, and left cardiac failure reported as the cause of death at Cycle 7. Non-drug-related TEAEs were reported as grade 4 pelvic haemorrhage for one subject, and cardiac arrest for one subject, both occurring at Cycle 2.

## DISCUSSION

Trabectedin, a novel marine-derived compound with a unique mechanism of action, has shown activity against a variety of preclinical solid tumour models. In a similar Phase II trial of trabectedin administered every 3 weeks in advanced ovarian cancer Sessa *et al* (2005) showed promising results with limited toxicities. Confirmation of these early results has been seen in the recently completed every 3 week regimen two arm randomised ET-743:B-026 study (Del Campo *et al*, 2006; McMeekin *et al*, 2007). The results of the present study and those reported by Sessa *et al* (2005), Del Campo *et al* (2006), and McMeekin *et al* (2007) demonstrate the effectiveness of trabectedin as a single agent in the platinum-sensitive patient population with respect to ORR and disease stabilisation. The most common TEAE in the three studies was a self-limiting reversible elevation in liver enzymes, with non-cumulative myelosuppression being a secondary common adverse event.

The efficacy results seen in all platinum-sensitive patients treated every 3 weeks with trabectedin in the SENDO study (Sessa *et al*, 2005) showed a response rate of 43.5% (95% CI: 23–65%) with TTP of 7.9 months (95% CI: 7.5–14.1 months). Similarly, the ET-743:B-026 study (Del Campo *et al*, 2006) showed a response rate of 37.4% (95% CI: 28.2–47.3%) with TTP of 6.8 months (95% CI: 5.5–7.4 months). These results trend favourably *vs* the response rate of 29% (95% CI: 18.2–41.9%) and TTP of 5.2 months (95% CI: 3.1–6.5 months) seen in this weekly trabectedin-treated schedule. However, differences in patient numbers as well as number of prior treatment regimens could contribute to the efficacy variations between the three studies.

In the case of platinum-resistant/refractory disease, response rates were low in this weekly schedule study (6.3% (95% CI: 2.1–14.2%), and less than that seen with the every 3 week trabectedin schedule (Sessa *et al*, 2005; Del Campo *et al*, 2006; McMeekin *et al*, 2007) where responses seen were 7% in all patients, and 14.3% (95% CI: 4.0–32.7%) in patients with one prior platinum-based regimen. The reduction in response observed in this study compared to the every 3 week data could be related to the inclusion of a higher percentage of third line and refractory patients in our study. The small but consistent number of durable responses seen in all three phase II trabectedin studies suggests that trabectedin could be combined with another active agent in this difficult to treat chemo-resistant patient population.

In contrast to the response rates, a more interesting finding in our study was the duration of response 5.2 months in platinum-sensitive and 4.2 months in platinum-resistant/refractory patient cohorts. The progression-free rate at 6 months was 44 and 11% for the platinum-sensitive and -resistant/refractory groups, respectively.

Trabectedin toxicity was manageable in this study, with only 7% of the patients discontinuing therapy due to a drug-related TEAE. Study related, regardless of association with drug, nausea (79%) and vomiting (56%) were common, although these were generally not severe (mostly grade 1 or 2) and did not limit therapy. Study related, regardless of association with drug, fatigue was reported in 70% of patients; however, the incidence of grade 3 (7%) or 4 (0%) fatigue was minimal. Grade 3 and 4 granulocytopaenia occurred much less frequently in the current trial (6%) compared with the Sessa trial (41%) (Sessa *et al*, 2005). The most common drug-related TEAE was dose- and schedule-dependent, self-limiting transaminitis (elevated, ALT 20%), which generally peaked by day 4 or 5 and returned to baseline by day 14.

In summary, trabectedin given as a single agent once weekly for 3 of 4 weeks is effective for the treatment of advanced relapsed ovarian cancer in the platinum-sensitive patient population. Responses in the heavily pretreated resistant/refractory patient population were low in both schedules (every week *vs* every 3 weeks). Myelosuppression appeared to be less in weekly schedule (6% *vs* 18%), whereas dose reductions and dose delays due to acute transaminitis was 42% in the weekly regimen, which is greater than the 34% reported in the every 3 week regimen (Sessa *et al*, 2005). Further studies in combination with effective and potentially synergistic agents in this patient population are ongoing. The chronicity of recurrent ovarian cancer suggests the development of more non-cross-resistant drug regimens for on-going palliative treatment. The reported results on tolerability, clinical efficacy, and durability demonstrate the potential usefulness of trabectedin as a novel single agent in advanced platinum-sensitive ovarian cancer in providing an alternative to existing single agents or combination treatments for ovarian cancer.

## Figures and Tables

**Table 1:** Demographics and baseline characteristics in treated patients

	**Platinum sensitive**	**Platinum resistant**	**Total**
	**(*N*=66)**	**(*N*=81)**	**(*N*=147)**
*Race, n* (%)			
*N*	66	81	147
Black	4 (6)	2 (2)	6 (4)
White	61 (92)	76 (94)	137 (93)
Asian	0	1 (1)	1 (1)
Other	1 (2)	2 (2)	3 (2)
			
*Age*			
*N*	66	81	147
Category, *n* (%)			
18–<40	3 (5)	2 (2)	5 (3)
40–<60	29 (44)	41 (51)	70 (48)
⩾60	34 (52)	38 (47)	72 (49)
Mean (s.d.)	60.1 (10.43)	58.3 (9.98)	59.1 (10.19)
Median	60.0	59.0	59.0
Range	(36; 83)	(33; 83)	(33; 83)
			
*Baseline weight in kilograms*			
*N*	66	81	147
Mean (s.d.)	71.58 (18.213)	69.15 (13.869)	70.24 (15.955)
Median	68.45	69.00	68.60
Range	(36.0; 167.0)	(42.3; 121.8)	(36.0; 167.0)
			
*Baseline ECOG Score, n* (%)			
*N*	66	81	147
0	46 (70)	47 (58)	93 (63)
1	20 (30)	34 (42)	54 (37)
			
*Histology, n* (%)			
*N*	66	80	146
Endometrioid	8 (12)	3 (4)	11 (8)
Clear cell carcinoma	2 (3)	5 (6)	7 (5)
Mixed epithelial tumour	1 (2)	2 (3)	3 (2)
Papillary/serous	49 (74)	59 (74)	108 (74)
Transitional carcinoma (Brenner)	1 (2)	0	1 (1)
Peritoneal carcinoma	1 (2)	2 (3)	3 (2)
Fallopian tube carcinoma	1 (2)	2 (3)	3 (2)
Other	3 (5)	7 (9)	10 (7)
			
*Histology grade, n* (%)			
*N*	66	79	145
Grade 1 (well- differentiated)	1 (2)	3 (4)	4 (3)
Grade 2 (moderately differentiated	16 (24)	12 (15)	28 (19)
Grade 3 (poorly differentiated)	44 (67)	56 (71)	100 (69)
Unknown	5 (8)	8 (10)	13 (9)
			
*Previous lines systemic therapy, n* (%)			
*N*	66	81	147
1 Prior platinum line	49 (74)	53 (65)	102 (69)
2 Prior platinum line	17 (26)	28 (35)	45 (31)

**Table 2:** Patient profile

	**Platinum sensitive**	**Platinum resistant**	**Total**
	**(N=66)**	**(N=81)**	**(N=147)**
**Reason for Withdrawal/Termination**	***n* (%)**	***n* (%)**	***n* (%)**
Total no. of subjects enrolled	66 (100)	81 (100)	147 (100)
			
Treated			
Evaluable for efficacy per RECIST Guidelines	62 (94)	79 (98)	141 (96)
Ongoing	3 (5)	3 (4)	6 (4)
Complete response (confirmed)	3 (5)	0	3 (2)
Adverse event/drug-related	6 (9)	2 (2)	8 (5)
Adverse event/not drug -related	2 (3)	6 (7)	8 (5)
Disease progression	42 (64)	57 (70)	99 (67)
Subject ineligible to continue	0	1 (1)	1 (1)
Subject choice	2 (3)	3 (4)	5 (3)
Other	4 (6)	7 (9)	11 (7)
			
Not evaluable for efficacy per RECIST Guidelines	4 (6)	2 (2)	6 (4)
Death	1 (2)	0	1 (1)
Adverse event/drug-related	0	1 (1)	1 (1)
Adverse event/not drug -related	1 (2)	0	1 (1)
Subject choice	2 (3)	1 (1)	3 (2)

Note: percentages calculated with the number of subjects in each group as denominator.

**Table 3:** Efficacy results

	**Platinum sensitive (*N*=66) *n* (%)**	**Platinum resistant (*N*=81) *n* (%)**	**Partial platinum sensitive (*N*=41) *n* (%) TTP=6 to <12 months**	**Platinum Sensitive (*N*=25) n (%) TTP=⩾12 months**
*Overall Best Response* a				
Evaluable	*N*=62 (100)	*N*=79 (100)	*N*=39 (100)	*N*=23 (100)
Complete response	4 (6)	0	1 (3)	3 (13)
Partial response	14 (23)	5 (6)	9 (23)	5 (22)
Stable disease	22 (35)	36 (46)	13 (33)	9 (39)
Not followed by PD	3 (5)	7 (9)	3 (8)	0
Followed by On-treatment PD	15 (24)	25 (32)	8 (21)	7 (30)
Followed by off-treatment PD	4 (6)	4 (5)	2 (5)	2 (9)
Progressive disease	22 (35)	38 (48)	16 (41)	6 (26)
Response rates	18 (29)	5 (6.3)	10 (26)	8 (35)
95% CI	(18.2; 41.9)	(2.1; 14.2)	(13.0; 42.1)	(16.4; 57.3)
				
*Progression-free survival* (*mo*)^b^				
Number of assessed	66	81	41	25
Number of censored	14 (21.2)	10 (12.3)	5 (12.2)	9 (36.0)
Number failed	52 (78.8)	71 (87.7)	36 (87.8)	16 (64.0)
Median (95% CI)	5.1 (2.8; 6.2)	2.0 (1.7; 3.5)	4.0 (1.7, 6.1)	5.1 (3.1; 6.5)
				
Overall survival (mo)^b^				
Number of assessed	66	81	41	25
Number of censored	49 (74.2)	37 (45.7)	29 (70.7)	20 (80.0)
Number failed	17 (25.8)	44 (54.3)	12 (29.3)	5 (20.0)
Median (95% CI)	— (14.8; —)^c^	11.1 (7.3; 13.0)	17.1 (14.8; —)^c^	— (11.5; —)^c^

Note: partial platinum-sensitive: TTP=6 to <12 months from end of last platinum-based treatment; Platinum-sensitive: TTP⩾12 months from end of last platinum-based treatment.

a All evaluable patients.

b Based on Kaplan–Meier product limit estimates.

c (—) Some medians, and lower and upper limits of confidence intervals not estimable.

**Table 4:** Worst on-treatment^a^ grade 1–4 laboratory abnormalities

**Lab type^b^**	**Grade 1 *n* (%)**	**Grade 2 *n* (%)**	**Grade 3 *n* (%)**	**Grade 4 *n* (%)**
Haematology				
Neutrophils	30 (21)	34 (23)	10 (7)	2 (1)
Haemoglobin	84 (58)	30 (21)	4 (3)	0
Platelets	18 (12)	4 (3)	4 (3)	0
				
Chemistry				
Hypoalbuminemia	38 (26)	37 (25)	10 (7)	0
Alk phos	44 (30)	4 (3)	3 (2)	0
AST (SGOT)	79 (54)	19 (13)	4 (3)	0
ALT (SGPT)	59 (40)	42 (29)	18 (12)	0
Bilirubin	8 (5)	4 (3)	0	0
Creatinine	21 (14)	0	0	1 (1)
Creatine Kinase	13 (9)	7 (5)	4 (3)	1 (1)
Hyperglycaemia	84 (58)	31 (21)	7 (5)	2 (1)
Hypoglycaemia	5 (3)	1 (1)	2 (1)	0
Hyperkalemia	8 (5)	2 (1)	2 (1)	0
Hypokalemia	43 (29)	0	6 (4)	0
Hypernatremia	13 (9)	0	0	0
Hyponatremia	46 (32)	0	11 (8)	1 (1)

a Worst grade between the first trabectedin dose and 30 days after the last trabectedin dose.

b For each lab test, *n*=146/147 treated patients for whom post-baseline lab data are available except for creatine kinase, hyperglycaemia, and hypoglycaemia in which cases *n*=145/147.

**Table 5:** Treatment-emergent drug-related grade 1–4 adverse events in ⩾2% of patients

		**All treated patients (*N*=147)**			
	**Total**	**Toxicity grade^a^**			
WHO preferred term	*n* (%)	1 (%)	2 (%)	3 (%)	4 (%)
SGPT (ALT) increased	41 (28)	6 (4)	20 (14)	14 (10)	1 (1)
Granulocytopaenia	35 (24)	9 (6)	17 (22)	7 (5)	2 (1)
Nausea	102 (69)	63 (43)	31 (21)	8 (5)	0
Vomiting	69 (47)	38 (26)	23 (16)	8 (5)	0
Fatigue	88 (60)	36 (24)	44 (30)	8 (5)	0
Gamma-GT increased	9 (6)	3 (2)	3 (2)	3 (2)	0
SGOT (AST) increased	21 (14)	8 (5)	10 (7)	2 (1)	1 (1)
Abdominal pain	16 (11)	7 (5)	6 (4)	3 (2)	0
Constipation	48 (33)	23 (16)	22 (15)	3 (2)	0
Creatine phosphokinase increased	7 (5)	2 (1)	2 (1)	2 (1)	1 (1)
Hypokalemia	4 (3)	1 (1)	0	3 (2)	0
Thrombocytopaenia	7 (5)	2 (1)	2 (1)	2 (1)	1 (1)
Somnolence	17 (12)	8 (5)	6 (4)	3 (2)	0

a Toxicity grade: NCI common terminology criteria, version 2.0. Incidence is based on the number of patients.
